# Use of suggestive seizure manipulation methods in the investigation of patients with possible psychogenic nonepileptic seizures—An international ILAE survey

**DOI:** 10.1002/epi4.12521

**Published:** 2021-07-31

**Authors:** Adrien Gras, Alistair Wardrope, Edouard Hirsch, Ali A Asadi Pooya, Rod Duncan, David Gigineishvili, Coraline Hingray, Kousuke Kanemoto, Lady Ladino, William Curt LaFrance, Aileen McGonigal, Chrisma Pretorius, Paola Valenti Hirsch, Pierre Vidailhet, Dong Zhou, Markus Reuber

**Affiliations:** ^1^ Liaison Psychiatry Unit 1 Place de l'Hopital University Hospitals Strasbourg Strasbourg France; ^2^ Academic Neurology Unit Royal Hallamshire Hospital The University of Sheffield Sheffield UK; ^3^ Department of Neurosciences Sheffield Teaching Hospitals NHS Foundation Trust Sheffield UK; ^4^ Epilepsy Unit "Francis Rohmer" INSERM Federation de Médecine Translationelle CHU‐University Strasbourg Strasbourg France; ^5^ Epilepsy Research Center Shiraz University of Medical Sciences Shiraz Iran; ^6^ Jefferson Comprehensive Epilepsy Center Department of Neurology Thomas Jefferson University Philadelphia PA USA; ^7^ Neurology Christchurch Hospital Christchurch New Zealand; ^8^ Department of Neurology and Neurosurgery Sarajashvili Institute of Neurology Tbilisi State University Tbilisi Georgia; ^9^ Department of Neurology Central Hospital Nancy Nancy France; ^10^ Neuropsychiatry Aichi Medical University Nagakute Japan; ^11^ Neurology Section Epilepsy Program Hospital Pablo Tobon Uribe Medellin Colombia; ^12^ Universidad de Antioquia Medellin Colombia; ^13^ Neuropsychiatry and Behavioral Neurology Rhode Island Hospital Providence RI USA; ^14^ Neurology and Psychiatry Brown University Providence RI USA; ^15^ Clinical Neurophysiology and Epileptology Department Hospital Timone Marseille France; ^16^ Institut de Neurosciences des Systèmes Aix‐Marseille Universite Marseille France; ^17^ Department of Psychology Stellenbosch University Stellenbosch South Africa; ^18^ Neurology Hautepierre Hospital University of Strasbourg Strasbourg France; ^19^ Fédèration de Medecine Translationelle Université de Strasbourg Strasbourg France; ^20^ Department of Neurology West China Hospital Sichuan University West China Hospital Chengdu China

**Keywords:** Psychogenic nonepileptic seizures (PNES), dissociative seizures, provocation methods, activation procedures, electroencephalography, suggestion, diagnosis

## Abstract

Video‐encephalographic (vEEG) seizure recordings make essential contributions to the differentiation of epilepsy and psychogenic nonepileptic seizures (PNES). The yield of vEEG examinations can be increased through suggestive seizure manipulation (SSM) (ie, activation/provocation/cessation procedures), but its use has raised ethical concerns. In preparation for guidelines on the investigation of patients with PNES, the ILAE PNES Task Force carried out an international survey to investigate practices of and opinions about SSM. An online questionnaire was developed by the ILAE PNES Task Force. Questions were asked at clinical unit or individual respondent level. All ILAE chapters were encouraged to send questionnaires to their members. The survey was open from July 1, 2019, to August 31, 2019. A total of 487 clinicians from 411 units across 94 countries responded. Some form of SSM was used in 296/411 units (72.0%). Over 90% reported the use of verbal suggestion, over 80% the use of activation procedures also capable of eliciting epileptic activity (hyperventilation or photic stimulation). Only 26.3% of units used techniques specifically intended to provoke PNES (eg, saline injection). Fewer than 10% of units had established protocols for SSM, only 20% of units required written patient consent, in 12.2% of units patients received explicitly false information to provoke seizures. Clinicians using SSM tended to perceive no ethical problems, whereas those not using SSM were likely to have ethical concerns about these methods. We conclude that the use of invasive nocebo techniques intended to provoke PNES in diagnostic settings has declined, but SSM is commonly combined with activation procedures also capable of eliciting epileptic activity. While research suggests that openness about the use of PNES‐specific nocebo techniques does not reduce diagnostic yield, very few units have suggestion protocols or seek patient consent. This could be addressed through establishing consensus guidance for the practice of SSM.

**Key points**

- Use of suggestive seizure manipulation (SSM) in diagnosing psychogenic nonepileptic seizures (PNES) has raised ethical concerns
- This survey describes current practice in and attitudes toward SSM in the diagnosis of PNES across 411 clinical practices in 94 countries
- SSM was used in 72% of units. Fewer than 10% of units had established protocols for SSM and only 20% required written patient consent
- Clinicians using SSM tended to perceive no ethical problems, whereas those not using SSM were likely to have ethical concerns
- This ILAE survey underscores the need for a standardized approach to the use of SSM in the diagnosis of PNES

## 1 INTRODUCTION

Psychogenic nonepileptic seizures (PNES) are a common problem in neurological practice, with an estimated prevalence of 50/100 000.^1^ The process leading to the diagnosis of PNES is often complex.^2^ The majority of patients are initially diagnosed with epilepsy, and the correct diagnosis of PNES can be delayed by several years.^3^ Twenty to 40 percent of patients admitted to tertiary centers turn out to have PNES rather than drug‐resistant epilepsy.^4^ The inappropriate prescription of antiseizure medications (ASMs) to patients with PNES erroneously diagnosed with epilepsy over long periods of time is associated with a high risk of iatrogenic harm (including death), and the provision of medical and social care to patients with PNES generates substantial costs to individuals, families, and society at large.^5^, ^6^ Delays in correct diagnosis also mean that appropriate and evidence‐based treatment of PNES is often delayed.

The confirmation of the diagnosis is the first step toward appropriate management (ie, stopping inappropriately prescribed ASMs and offering psychotherapeutic care). Correct diagnosis provides the basis for an effective delivery of an explanation of the condition to patient and family, which can be considered as a therapeutic intervention in itself^7^ and which has been shown to lead to the cessation of PNES in at least one in six patients.^8^

The Psychogenic Nonepileptic Seizures Task Force of the International League Against Epilepsy (ILAE) delineated a staged approach to PNES diagnosis.^9^ Although descriptions of seizure manifestations by patients and witnesses make a very important contribution to the diagnosis of PNES, the highest level of diagnostic certainty can only be reached through the simultaneous video‐ and EEG‐recording of a typical seizure event.^9^, ^10^, ^11^, ^12^

In view of the diagnostic gain the recording of a typical PNES can achieve, clinicians have attempted to increase the yield of EEG or video‐EEG (vEEG) recordings by using suggestive seizure manipulation (induction and cessation) (SSM) methods, also known as seizure provocation techniques.^13^ For the purposes of this paper, the term SSM includes any procedure used to make the occurrence of a PNES more likely, ranging from simple verbal suggestion to the intravenous injection of placebo. Commonly reported SSM methods also include conventional EEG activation procedures like hyperventilation, intermittent photic stimulation, temporal compression, or a combination of these techniques.^14^

The use of suggestion as a diagnostic method in this context dates at least as far back as Jean‐Martin Charcot and the Salpêtrière School in Paris, France, during the 1880s. In his clinical lessons that attracted large audiences, Charcot used a range of provocation methods, including hypnosis to trigger “hystero‐epilepsy.”^15^ Charcot also encouraged efforts to capture the visible seizure semiology in drawings and photographs.^16^ The role of the EEG in the distinction of epileptic and nonepileptic seizures was recognized soon after the initial description of this physiological measure by Berger in 1929.^17^ The importance of filming of seizures for diagnostic and research purposes was highlighted by Loewenstein in 1933, and in 1945, Herbert Kupper first described the use of SSM for PNES diagnosis during an EEG‐recording.^18^

Although it is not possible to report a mean additional diagnostic yield rate achieved by SSM techniques in view of the heterogeneity of patient selection and SSM methods in the primary research, more recent studies have confirmed the potential of SSM to be associated with improvements in the diagnostic yield of time‐limited (v)EEG recordings in patients with suspected PNES in both inpatient and outpatient settings. This research has been summarized recently in a systematic review.^14^ SSM methods have, however, increasingly been subject to controversy regarding their clinical relevance, acceptability to patients, ethical issues, and potential impact on the doctor‐patient relationship.^19^, ^20^ Nevertheless, relatively recent surveys confirmed the widespread use of SSM techniques. For instance, two surveys conducted in the United States in 1996 revealed that 40% of neurologists routinely used SSM^21^ and that SSM was used in 73% of epilepsy centers.^22^ We are not aware of any previous studies examining the worldwide use of SSM in this clinical context, but publications from many countries demonstrate that these techniques have been used more recently and not only in the United States.^14^

In preparation for future ILAE guidance on diagnostic procedures for suspected PNES and in close collaboration with the ILAE PNES Task Force, we created an online questionnaire that we sent to Epilepsy Units around the world, through the chapters of the ILAE. The aims of the survey about the diagnostic use of SSM techniques in the context of suspected or possible PNES were to:

1. establish the frequency of use and nature of SSM practices around the world;
2. explore the concomitant use of (v)EEG recordings;
3. collect information about patient information and consent procedures;
4. record diagnostic criteria used in the evaluation of seizure recordings;
5. gather information on adverse effects, seizure management, acceptability, patient feedback; and
6. learn about the personal views of clinicians in relation to utility, acceptability, ethical concerns, impact on patient‐doctor relationship

By answering these questions, we hope to establish the extent of SSM use in clinical practice, variation in its use, and scope for improving clinical practice through standardization of SSM use, as well as attitudes of relevant stakeholders to SSM, in line with ILAE recommendations on clinical practice guideline development.^23^

## 2 METHODS

### 2.1 Questionnaire development

The aims of this project and the questionnaire used for data collection were developed iteratively and in close collaboration between a French group of epileptologists and psychiatrists who initiated this project and the ILAE PNES Task Force. Earlier drafts of the survey were tested and improved by the international members of the PNES Task Force to ensure that all questions were understandable and relevant in different cultural and healthcare system contexts. The questionnaire used in this survey is available as additional online web content (see Appendix S1). It comprised demographic and unit information (9 multiple‐choice questions [MCQs]) and summary of unit‐level practice (4 MCQs for those working in units where SSM had never been used, 6 in units where it was previously, but no longer used, and 8 MCQs and 6 binary yes/no questions where SSM is still used). For those working in units where SSM is used, there were additional sections: a summary of procedures used (19 ordinal‐scale questions, 2 binary yes/no questions, 1 MCQ) and a survey of personal attitudes toward SSM (3 ordinal‐scale frequency questions, 16 Likert‐scaled opinion questions).

### 2.2 Respondents

A link to an online Google Forms^®^ version of the questionnaire (in English) was distributed by the ILAE to all of its national chapters with the request that chapters should disseminate it further to all of their professional members at the start of June 2019. ILAE members were also encouraged to complete the survey via Epigraph, the ILAE’s regular online news magazine for members. In total, the ILAE currently has over 15 000 members (although we cannot be certain that the invitation email reached all members). A reminder was sent to all chapters with a request to pass it on to their members in June 2019. The survey was closed to further replies in August 2020. For illustrative purposes, Epigraph's most recent circulation was 11 164. Of these, 2139 (19.16%) were opened.

### 2.3 Statistical analysis

Prior to analysis, we re‐coded data for questions referring to institutional‐level practice to account for multiple respondents from the same institution; where multiple respondents from the same institution gave different responses, we used either the maximum, minimum, or median of all responses from that institution, depending on the question (eg, for questions referring to frequency of use we took the median response, whereas for questions asking whether a certain technique is ever used, a single positive answer would outweigh other negative answers). We summarized binary data as a proportion of respondents and scale data by identifying the response median (for central tendency) and interquartile range IQR (for spread).

We used Friedman's two‐way ANOVA by rank to assess differences in the frequency of use of induction and cessation tests with post hoc Mann‐Whitney U tests using a Bonferroni‐corrected α = 0.05 to test significance of pairwise comparisons if the null hypotheses of the ANOVA were rejected.

We compared attitudes toward verbal suggestion and activation procedures against intravenous (IV) induction using Wilcoxon's test, with Bonferroni's correction for multiple comparisons. In order to gain a more fine‐grained understanding of the responses, we carried out a number of exploratory statistical analyses without prespecified hypotheses (ie, differences in attitudes toward SSM use depending on career background, gender, and age) using Kruskal‐Wallis or Mann‐Whitney U tests, or ordinal regression, as appropriate. In order to examine whether other ethically contentious practices within units would affect respondents' attitudes to SSM, we tested for differences in attitudes toward SSM between those working in units who did or did not give explicitly false information to patients prior to SSM, and did or did not seek explicit consent for SSM (Mann‐Whitney U test, Bonferroni adjustment for multiple comparisons).

All statistical analyses were performed in IBM SPSS Statistics v26.0.0.0 (IBM Corp, Armonk NY).

### 2.4 Regulatory approval

The final version of the questionnaire was reviewed by the Psychiatry Commission and approved for dissemination via the national chapters by the Executive Committee of the ILAE. While requirements regarding ethical review for survey research may differ internationally, independent ethical review was not required for anonymous survey of members of a professional organization in the countries in which the leaders of this project were based (France and United Kingdom). According to current best practice in the ethical review of survey research, this project would not require institutional ethics oversight, given that it did not involve vulnerable subjects and posed low risk of informational or psychological harm to respondents.^24^

## 3 RESULTS

### 3.1 Participant demographics

**FIGURE 1:**
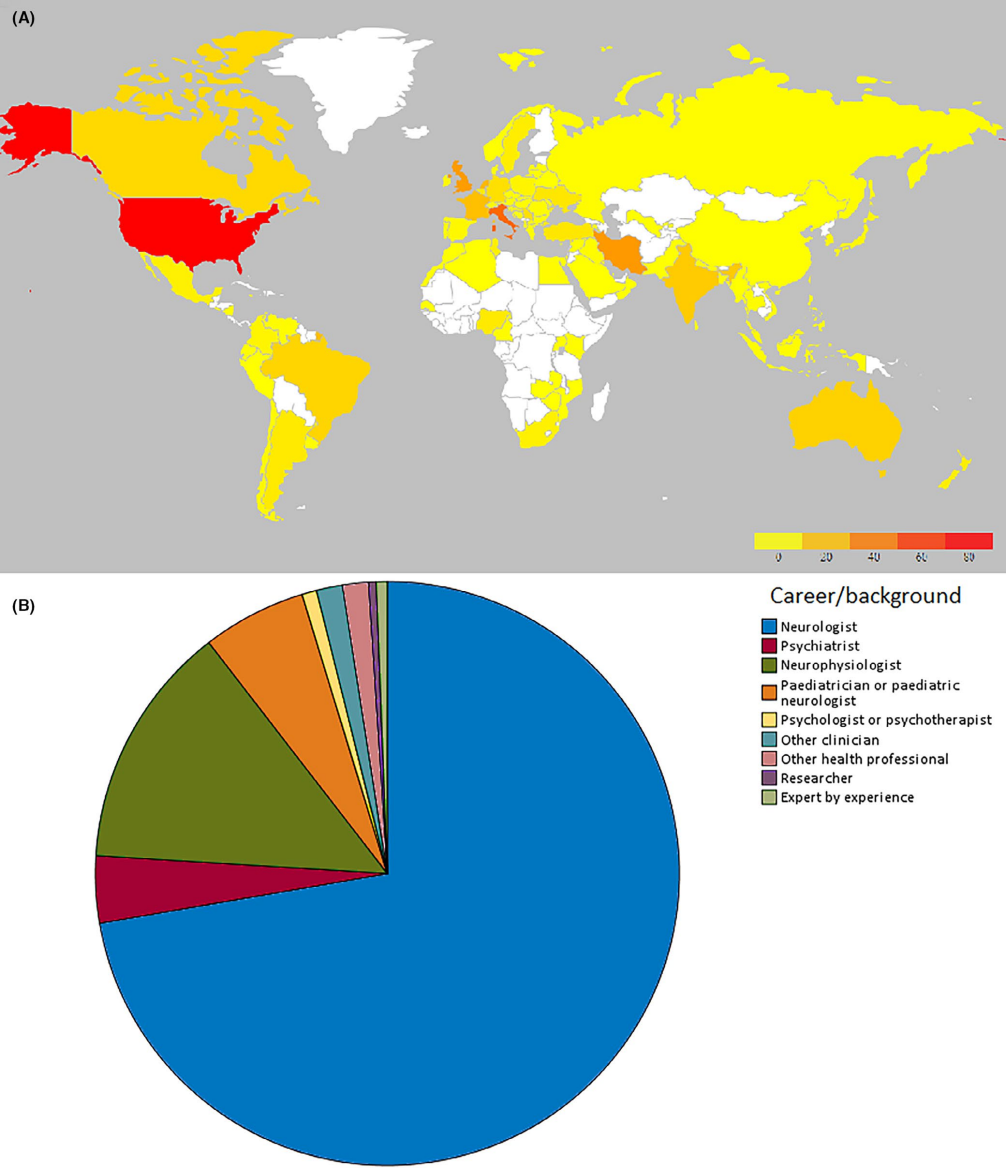
Geographic and career background distribution of respondents. (A) Heat map showing global distribution of respondents (B) career background of respondents

A total of 487 respondents completed the survey. As the survey was distributed openly to ILAE members through national chapters and online publications, we are unable to determine the exact number of individuals invited to participate and as such, in the absence of a denominator cannot determine a response rate. Respondents came from 411 different units across 94 countries (see Figure 1A). The majority of respondents were neurologists or epileptologists (72.3%); the remainder comprised other clinicians (24.4%), other health professionals (2.2%), and researchers or experts by experience (see Figure 1B). 218 respondents (44.8%) were female. Participant ages ranged from <30 years to >70 years, median category 40‐50 years old.

### 3.2 Findings at unit‐level

#### 3.2.1 Usage rates

**FIGURE 2:**
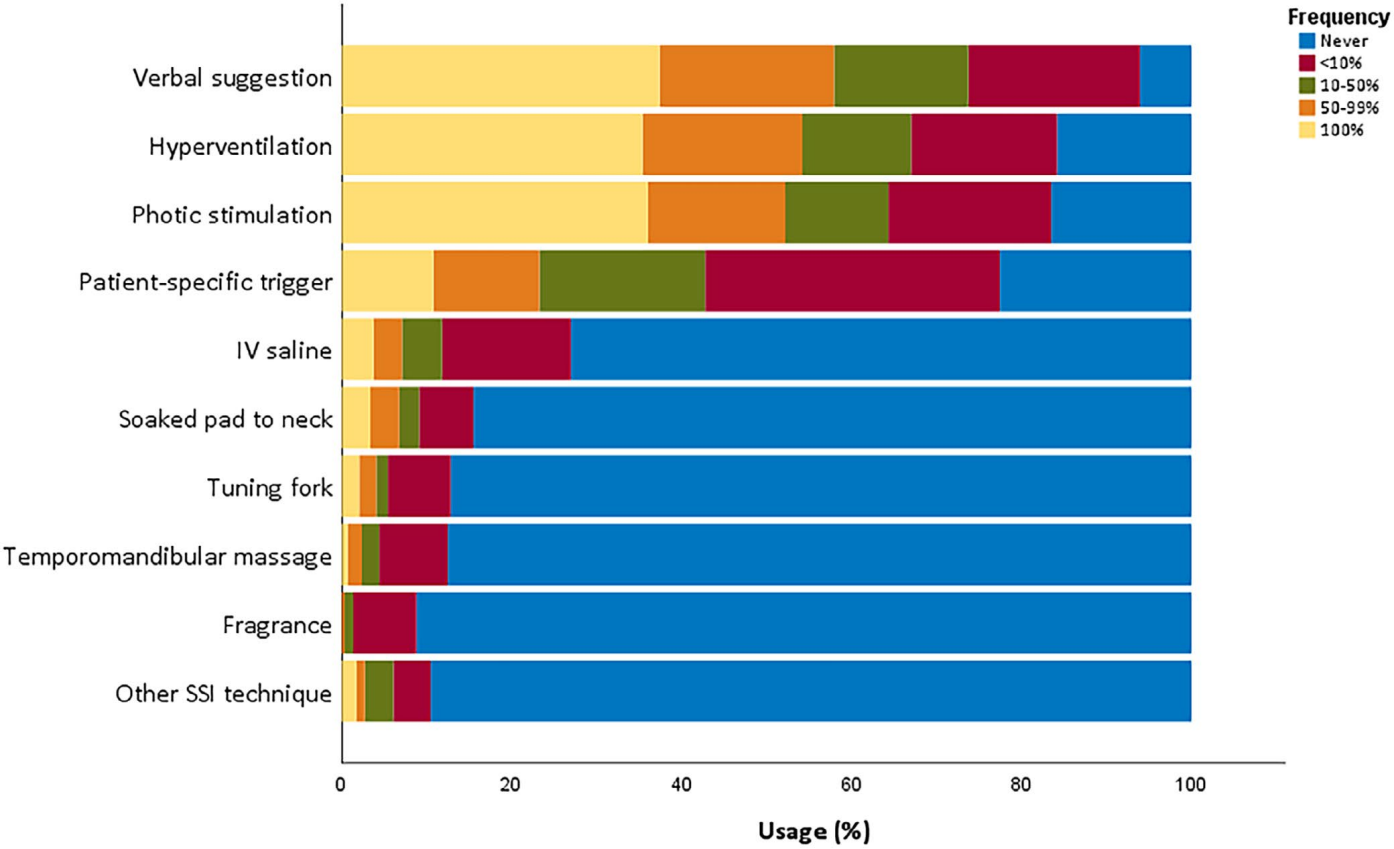
Frequency of SSM technique use

Some form of SSM was used in 296/411 units (72.0%). The most commonly used SSM technique was verbal suggestion alone (VS; at least sometimes used in 93.9% of those centers where any SSM is used at all), followed by activation procedures also capable of eliciting epileptic activity such as hyperventilation (HV; 84.2%) and photic stimulation (PS; 83.5%). See Figure 2 for frequency of use of different SSM techniques and Table S1 for additional information. Despite previously being considered a standard provocation technique,^25^, ^26^ induction by nocebo techniques specifically intended to elicit PNES such as intravenous (IV) injection or infusion (usually saline) were used markedly less frequently, with 73.1% of responding units reporting that they were *never* used. Other less common techniques reported to be in use include the following: administration of nocebo by different routes (oral, subcutaneous, transdermal; 7 respondents); auditory stimulus (eg, music, sudden loud noise; 3 respondents); tactile stimulus (3 respondents); hypnotic suggestion (2 respondents); exercise (2 respondents); review of recordings of previous events; warm environment; and tilt‐table testing (all one respondent).

Frequency of use of different SSM techniques differed significantly (Friedman's two‐way ANOVA by rank, *df* = 9, *P <* .001). Verbal suggestion, hyperventilation, and photic stimulation were all used more frequently than other techniques, including IV saline induction (IVI). The frequency of VS, HV, and PS did not differ significantly in pairwise comparisons (Mann‐Whitney *U*, Bonferroni‐adjusted α = 0.05).

At the unit level, the most frequently cited reasons for not using SSM were as follows: ethical concerns (34.8%); false‐positive risk (28.7%); damage to the doctor‐patient relationship (24.3%); and SSM being unnecessary (24.3%).

Not all units' respondents answered all questions relating to the use of suggestion to stop seizures once in progress. 242/286 (84.6%) reported sometimes using VS, 92/277 (33.2%) IVI, and 38/227 (16.7%) other techniques. The frequency of use of different suggestive seizure cessation techniques differed significantly (Friedman's two‐way ANOVA by rank, *df* = 2, *P* < .001). VS was used significantly more frequently than IVI or other techniques (Mann‐Whitney *U*, Bonferroni‐adjusted *P* < .001).

#### 3.2.2 SSM protocols

**FIGURE 3:**
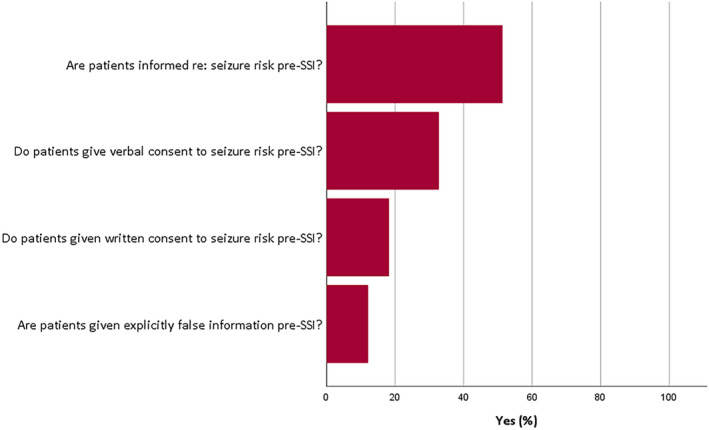
Use of information and consent procedures

The responses suggested that 263 units (88.9%) did not have a written protocol for SSM. In 75 units (25.3%), SSM was “sometimes” performed without EEG monitoring. Patients were informed of the “risk of triggering a seizure” in only 152 (51.4%) of units and gave written consent in 54 (18.2%). In 36 units (12.2%), patients were given explicitly false information pre‐SSM (see Figure 3).

#### 3.2.3 SSM risks

In 92 centers (31.1%), SSM was reported as having induced “false positive” epileptic seizures; in 81 centers (27.4%), SSM induced prolonged PNES requiring admission. Our survey did not record quantitative estimates of the frequency of these adverse events.

Negative comments from patients post SSM were reported by responses from 93 (33.3%) of centers, whereas 213 (81.9%) reported positive comments.

### 3.3 Findings at individual respondent level

#### 3.3.1 Attitudes in units where SSM is used

**FIGURE 4:**
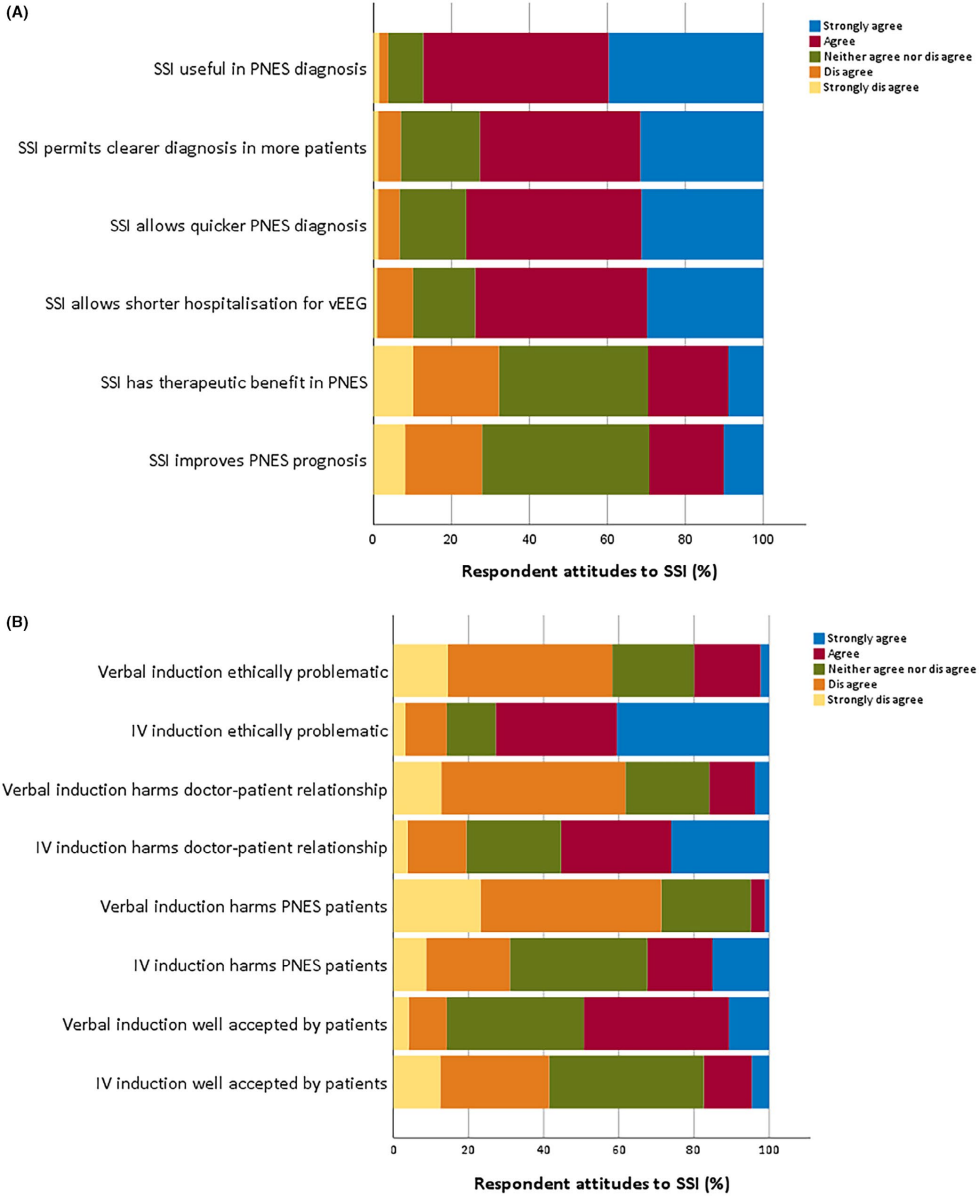
Respondent attitudes to SSM. (A) respondent attitudes to utility of SSM; (B) attitudes to ethics of SSM

A total of 342/487 (70.8%) of respondents worked in institutions where SSM was at least sometimes used. Most had favorable opinions regarding the utility of SSM, either agreeing or strongly agreeing that SSM is useful in diagnosing PNES (87.2%); allows clearer diagnosis in more PNES patients (72.8%); permits quicker diagnosis in more PNES patients (76.2%); and allows shorter hospitalization for vEEG (74.0%). Respondents were more equivocal concerning whether SSM had therapeutic benefit in PNES (29.6% agree v. 32.1% disagree) or improved PNES prognosis (29.2% agree v. 27.8% disagree). Responses are portrayed graphically in Figure 4A.

Most individual respondents using SSM felt they personally were honest in explaining the procedure to patients (median 8, IQR 5‐9 on a 10‐point scale where 1 is actively misleading and 10 fully honest). 10.4% of respondents worked in institutions where they were encouraged to give explicitly false information regarding SSM to patients, and these respondents rated their explanations as significantly less honest than respondents from institutions where they were not so encouraged (median [IQR] rating of honesty 5 [2.5‐8] in institutions where explicitly false information encouraged v 8 [5.25‐9] where it was not, *P* < .001).

Similarly, the majority of respondents from units where it is used felt comfortable with performing SSM (median 8, IQR 5‐9, where 1 is very uncomfortable and 10 fully comfortable). Respondents from these units were significantly less concerned regarding the ethics of VS than IVI for SSM. Respondents felt that IVI was more ethically problematic, harmed the doctor‐patient relationship more, harmed people with PNES more, and was less well‐accepted by patients (Wilcoxon test; Bonferroni‐adjusted *P* < .001 for all, see Figure 4B and Table S2 for summaries of respondents' ethical concerns).

Attitudes toward SSM did not significantly vary (Bonferroni‐corrected α = 0.05) between respondents characterized by different: career backgrounds (Kruskal‐Wallis test); genders (Mann‐Whitney *U*); or ages (ordinal regression, Probit link function).

Respondents who sought explicit consent to SSM were less likely to agree that verbal induction harmed patients with PNES (Mann‐Whitney *U*, *P* = .001). Otherwise, attitudes did not vary with consent procedures. Respondents who gave explicitly false information to patients undergoing SSM did not significantly differ in their answers to the eight questions on the ethics of SSM (Figure 4B) from those who did not.

#### 3.3.2 Attitudes in units where SSM is not used

A total of 142/487 (29.2%) of respondents worked in units where SSM is not used. The majority (78.2%) of respondents working in units where SSM is not used felt there were potential ethical or legal problems with SSM, most commonly that it violates patient trust (51.4%), damages the doctor‐patient relationship (44.4%), or violates informed consent (43.0%, see Table S3 for further details).

## 4 DISCUSSION

### 4.1 Use of SSM

We found that the majority (72%) of epilepsy units worldwide made use of SSM in some form—in the majority of cases, conventional activation procedures. This figure appears consistent with usage rates reported in previous surveys conducted in the United States.^22^ Noninvasive techniques (HV, PS, VS) and techniques which could induce epileptic activity as well as PNES are used more frequently than provocation techniques specifically intended to provoke PNES, such as IV nocebo administration. This is despite the fact that most published research on the effectiveness of SSM in diagnosis of PNES uses invasive nocebo administration.^14^ This may reflect recent evidence of the noninferiority of SSM without nocebo administration,^27^, ^28^, ^29^ or it may be related to increasing ethical concerns of using PNES‐specific invasive provocation techniques (discussed below). However, it is important to note that in one recent study in which noninvasive techniques were used prior to nocebo injection, PNES were only captured in 12/27 (44%) patients after saline injection.^30^

There was notable heterogeneity regarding the use of SSM between and within responding units. The four most commonly employed SSM techniques (HV, PS, VS, and patient‐specific triggers) were all deployed with a wide range of frequencies across units, and the majority of units would only utilize these in some patients and not others. This may reflect the previously observed lack of general consensus and therefore clear guidelines for the standardized employment of SSM within routine EEG,^31^ a fact underlined by the vast majority of responding units not having written protocols for SSM usage.

### 4.2 Attitudes toward SSM usage

The majority of respondents from units where SSM is used were comfortable with its performance, though the majority had greater concerns regarding nocebo induction than VS. These concerns related more to the ethics of nocebo induction than potential harms, with many respondents agreeing that provocative techniques may either be ethically problematic (unfortunately the survey design of our research prevented interrogating this further) or might harm the doctor‐patient relationship. There is some evidence that SSM protocols without invasive techniques such as IV nocebo are noninferior to invasive ones (ie, statistically as likely to be diagnostically helpful).^27^ However, since several conventional noninvasive techniques may also reduce the threshold for epileptic seizures (especially hyperventilation and photic stimulation), there may remain contexts where the risks of an epileptic seizure are felt sufficiently high to make alternative methods of nocebo induction preferable (one such context which has been cited is the late stage of pregnancy).^32^ If nocebo induction is used, it will be important to mitigate ethical concerns. This could be done by using disclosure and informed consent, along with protocols that honestly inform patients of the placebo nature of the induction technique and of the possibility that PNES may occur. While concerns persist that this may reduce the diagnostic yield, published research suggests that explicit, informative protocols do not decrease (and may increase) the diagnostic yield of SSM.^28^, ^30^, ^33^, ^34^ A first step in addressing this issue would be to stop (or minimize) deliberately misinforming patients, reported in 12.2% of units. Explicit guidelines on ethical use of SSM would empower practitioners to employ SSM in a more responsible fashion^31^ and would not be expected to reduce diagnostic yield.^14^

### 4.3 Limitations

Several limitations to this study should be noted. It is difficult to ascertain how representative our sample of respondents is of the community of epilepsy professionals. Despite many responses from a wide range of countries spanning six continents, given the manner in which the survey was circulated (with ILAE national chapters being responsible for dissemination to their own members), we cannot accurately determine a response rate. The survey was only available in English, which may bias the results toward those regions where English is a main spoken or professional language. Not all health workers involved in diagnosis of PNES—including SSM usage—will be ILAE members, introducing a further potential source of selection bias. Differential response rates to survey invitations between specialties may unduly weight our responses further toward those from neurologists.^35^

The use of different questionnaires for respondents working in units where SSM is or is not routinely used precluded a direct comparison of attitudes toward SSM between respondents working in these different settings. This would be a question of interest in attempting to design protocols for SSM usage, to ensure the concerns of those who do not presently make use of such techniques are addressed appropriately.

While the survey documented reports of adverse effects of SSM (such as “false positive” epileptic seizures or prolonged PNES), we did not ask respondents to report frequency of these events, so cannot reliably conclude how often these commonly cited adverse effects of SSM occur. Such figures would clearly be of use in appraising benefits and risks of SSM and seeking fully informed consent from patients.

The use of a survey design itself introduces some important limitations. Forced‐response choices limit fine‐grained analysis of our respondents' attitudes toward SSM usage, which would more appropriately be interrogated with qualitative research.^36^ Such research would be a valuable complement to our results; however, the present survey permits a far larger sample than could be achieved in such a study and thus is able to present a more representative picture of international clinicians’ use of and attitudes toward SSM.^37^

Lastly, our survey was addressed to healthcare workers and not patients with seizure disorders, and thus, few conclusions can be drawn about the acceptability and tolerability of SSM to patients. However, our respondents' reported low rates of dissatisfaction with SSM that are supported by previous research suggesting that patients generally find SSM acceptable and comfortable and are willing to undergo them when their use is explained properly.^38^

## 5 CONCLUSION

Suggestive seizure manipulation, in the forms of conventional EEG activation procedures (hyperventilation and photic stimulation) and verbal suggestion, is a widespread technique used in the diagnosis of PNES, with proven value in increasing the diagnostic yield of (v)EEG. However, the employment of SSM is not at all standardized within and between epilepsy units, potentially reducing the efficiency and reliability of these investigations. Furthermore, though not widespread, more invasive SSM provocation techniques specifically intended to provoke PNES (eg, iv saline infusion) are still sometimes used in a deceptive or otherwise ethically suspect fashion.

The findings of this ILAE survey underscore the need for a standardized approach to the use of SSM in the diagnosis of PNES. While proposals for best practice exist,^31^ there are as of yet no internationally accepted standardized guidelines, and many units lack even internal protocols for SSM. This study should stimulate different EEG laboratory units worldwide to share their experience and start discussion about standardization of SSM procedures for making accurate diagnosis of PNES. Further research involving a more nuanced qualitative approach and capturing the views of patients and clinicians would be useful to inform the development of guidelines for the safe and ethical use of SSM. In the interim, departments could use recent summaries of evidence surrounding use of SSM,^14^ and previous proposals for best practice guidance on its use,^31^ as templates for developing local protocols to ensure safe, ethical, and consistent use of this technique in clinical practice.

## CONFLICT OF INTEREST

This report was written by experts selected by the International League Against Epilepsy (ILAE) and was approved for publication by the ILAE. Opinions expressed by the authors, however, do not necessarily represent the policy or position of the ILAE. None of the authors has conflicts of interest to disclose relevant to this manuscript. We confirm that we have read the Journal's position on issues involved in ethical publication and affirm that this report is consistent with those guidelines.

## Supporting information

Appendix S1
