# Supplementary material for: Use of suggestive seizure manipulation methods in the investigation of patients with possible psychogenic nonepileptic seizures—An international ILAE survey
Source: Epilepsia Open. 2021 Jul 31;6(3):472–82. doi: 10.1002/epi4.12521 (PMC8408588; doi:10.1002/epi4.12521)
Supplement: Supplementary file 1 — Appendix S1 [file EPI4-6-472-s001.docx]

## Supporting Table 1: Frequency of SSM technique use *(additional online content)*

| Technique | Frequency, *N* (%) | | | | |
| --- | --- | --- | --- | --- | --- |
|  | **Never** | **<10%** | **10-50%** | **51-99%** | **Always** |
| Verbal suggestion | 18 (6.1) | 60 (20.2) | 47 (15.8) | 61 (20.5) | 111 (37.4) |
| IV saline | 217 (73.1) | 45 (15.2) | 14 (4.7) | 10 (3.4) | 11 (3.7) |
| Hyperventilation | 47 (15.8) | 51 (17.2) | 38 (12.8) | 56 (18.9) | 105 (35.4) |
| Photic stimulation | 49 (16.5) | 57 (19.2) | 36 (12.1) | 48 (16.2) | 107 (36.0) |
| TMJ massage | 260 (87.5) | 24 (8.1) | 6 (2.0) | 5 (1.7) | 2 (0.7) |
| Soaked pad to neck | 251 (84.5) | 19 (6.4) | 7 (2.4) | 10 (3.4) | 10 (3.4) |
| Fragrance | 271 (91.2) | 22 (7.4) | 3 (1.0) | 1 (0.3) | 0 (0.0) |
| Patient-specific trigger | 67 (22.6) | 103 (34.7) | 58 (19.5) | 37 (12.5) | 32 (10.8) |
| Other technique | 266 (89.6) | 13 (4.4) | 10 (3.4) | 3 (1.0) | 5 (1.7) |

## Supporting Table 2: Attitudes of SSM users toward the ethics of SSM use *(additional online content)*

| Do you agree? | Frequency, *N* (%) | | | | |
| --- | --- | --- | --- | --- | --- |
|  | **Strongly agree** | **Agree** | **Neither agree nor disagree** | **Disagree** | **Strongly disagree** |
| VS ethically problematic | 8 (2.3) | 61 (17.7) | 75 (21.7) | 151 (43.8) | 50 (14.5) |
| IVI ethically problematic | 140 (40.6) | 111 (32.2) | 45 (13.0) | 38 (11.0) | 11 (3.2) |
| VS harms doctor-patient relationship | 13 (3.8) | 42 (12.2) | 77 (22.3) | 169 (49.0) | 44 (12.8) |
| IVI harms doctor-patient relationship | 90 (26.1) | 101 (29.3) | 87 (25.2) | 54 (15.7) | 13 (3.8) |
| VS harms PNES patients | 4 (1.2) | 13 (3.8) | 82 (23.8) | 166 (48.1) | 80 (23.2) |
| IVI harms PNES patients | 52 (15.1) | 60 (17.4) | 126 (36.5) | 77 (22.3) | 30 (8.7) |
| VS well accepted by PNES patients | 37 (10.7) | 133 (38.6) | 126 (36.5) | 35 (10.1) | 14 (4.1) |
| IVI well accepted by PNES patients | 16 (4.6) | 44 (12.8) | 142 (41.2) | 100 (29.0) | 43 (12.5) |

## Supporting Table 3: Attitudes of SSM non-users toward the ethics of SSM *(additional online content)*

| Potential ethical or legal problem for SSM | Agree (N, %) | Disagree (N, %) |
| --- | --- | --- |
| Informed consent | 61 (43.0) | 81 (57.0) |
| Violation of patient trust | 73 (51.4) | 69 (48.6) |
| Damage to the doctor-patient relationship | 63 (44.4) | 79 (55.6) |
| Damage to community trust | 24 (16.9) | 118 (83.1) |
| Disrespecting patient autonomy | 35 (24.6) | 107 (75.4) |
| Induction-related harm | 21 (14.8) | 121 (85.2) |
| No problem | 31 (21.8) | 111 (78.2) |
